# A Case of Dislocation of the Hand and Carpus Backwards

**Published:** 1899-06

**Authors:** Trafford Mitchell


### A CASE OF DISLOCATION OF THE HAND AND CARPUS BACKWARDS.

BY

TRAFFORD MITCHELL, M.D. Glasg.

On 23rd August I was called to see M. J., a boy aged 13 years. Whilst crossing an earthen embankment five feet high on his way to fetch water, his foot had caught in a wire on the top of the embankment, and he had fallen to the ground with his right hand under him, the back of the hand being downwards. When he got up again he found himself unable to catch hold of the tin vessel he had been carrying. He then returned home, and fainted on account of the pain. I found both bones of the right forearm perfectly sound, of normal shape, and free from crepitus; but on the dorsal aspect I found an abrupt projection, which, I soon discovered, was due to the carpus overlapping the lower end of the radius, whilst the lower part of the latter bone projected on the palmar aspect. There was shortening of the limb below the elbow. By traction on the hand and gentle pressure on the carpus, all deformity disappeared, and the pain immediately ceased. There were a few abrasions on the back of the hand, and a little discolouration was subsequently visible in the neighbourhood of the wrist. The treatment employed consisted only of the application of a little liniment and a narrow flannel bandage, splints being found unnecessary. The wrist is now as strong as ever, and free from deformity.

